# Radiological and Histopathological Correlative Analysis of Bone Tumors and Tumor-Like Lesions at a Tertiary Care Center

**DOI:** 10.7759/cureus.110277

**Published:** 2026-06-04

**Authors:** Neha Kumari, Manoj K Paswan, Abhishek Guria, Sunil Kumar Mahto, Deepali Tirkey, Anshu Jamaiyar

**Affiliations:** 1 Pathology, Rajendra Institute of Medical Sciences, Ranchi, IND; 2 Orthopedics, Rajendra Institute of Medical Sciences, Ranchi, IND; 3 Ophthalmology, Rajendra Institute of Medical Sciences, Ranchi, IND

**Keywords:** bone tumors, concordance, histopathology, radiology, tumor-like lesions

## Abstract

Background: Bone tumors and tumor-like lesions represent a heterogeneous group of disorders with overlapping clinical and radiological characteristics, often posing diagnostic challenges in routine practice. The present study aimed to evaluate the spectrum of bone tumors and tumor-like lesions encountered at a tertiary care hospital in eastern India and to assess the concordance between radiological and histopathological diagnoses. Materials and methods: This prospective observational study was conducted in the Department of Pathology over 18 months and included 125 patients with clinically and radiologically suspected bone lesions who underwent biopsy or surgical intervention. Radiological findings were correlated with histopathological examination, and concordance was assessed using Cohen’s kappa statistic. A p-value <0.05 was considered significant. Results: Most patients were below 30 years of age (98, 78%), with male predominance (72, 58%). Swelling associated with pain (48, 38%) was the most common presenting symptom, and the femur was the most frequently involved bone. Radiological evaluation categorized 52 (42%) lesions as malignant, whereas histopathology identified malignancy in 47 (38%) cases. Osteosarcoma was the most common malignant tumor. Overall concordance between radiological and histopathological diagnoses was observed in 106 (85%) cases, with a kappa value of 0.636 indicating statistically significant moderate agreement. Conclusions: Radiological evaluation demonstrated moderate agreement with histopathological diagnosis and served as an effective preliminary diagnostic modality in bone tumors and tumor-like lesions. However, histopathological examination remained essential for a definitive diagnosis because of overlapping imaging features among benign, aggressive, and malignant lesions.

## Introduction

Bone is a specialized connective tissue that supports the body, aids locomotion, safeguards internal organs, and is essential in mineral homeostasis and hematopoiesis [1]. Due to its complicated structure and cellular dynamics, bone is prone to a broad range of pathological conditions, including inflammatory and metabolic disorders as well as neoplastic and tumor-like lesions [1]. Bone lesions may present in varied clinical forms, with some showing rapid onset of pain and swelling, while others remain asymptomatic and are detected incidentally on imaging [2]. Many of these conditions share overlapping clinical and radiological features, making accurate diagnosis challenging in routine practice [3]. Bone tumors are broadly classified as benign or malignant, with further classification into primary and secondary lesions. Benign tumors such as osteochondroma, enchondroma, and osteoid osteoma typically exhibit localized growth without metastatic potential, although some may behave aggressively [4]. Malignant tumors, including osteosarcoma, Ewing sarcoma, and chondrosarcoma, demonstrate destructive growth patterns with metastatic potential and often carry a poor prognosis if not identified early [5]. Secondary tumors form a substantial proportion of malignant bone lesions in older individuals. Tumor-like lesions include aneurysmal bone cyst, simple bone cyst, fibrous dysplasia, non-ossifying fibroma, eosinophilic granuloma, and osteomyelitis-related reactive lesions, which may closely resemble true neoplasms clinically and radiologically, posing diagnostic difficulties [6].

Epidemiologically, bone tumors are rare, accounting for approximately 0.5% of all cancers worldwide and about 0.9% of neoplasms in Indian hospital-based studies, with a male predominance and a bimodal age distribution [7]. Radiological imaging serves as the basis for initial evaluation; however, histopathological examination remains the gold standard for definitive diagnosis [8-10]. Accurate interpretation requires correlation of clinical, radiological, and pathological findings, as discrepancies among these modalities have been documented [11]. The present study aimed to evaluate the spectrum of bone tumors and tumor-like lesions encountered at a tertiary care hospital in eastern India and to assess the concordance between radiological and histopathological diagnoses.

## Materials and methods

After obtaining permission from the Institutional Ethics Committee of Rajendra Institute of Medical Sciences (approval number: 65, dated February 1, 2025), this prospective observational study was conducted in the Department of Pathology at the Rajendra Institute of Medical Sciences over a period of 18 months, from August 2024 to January 2026. All participants were informed beforehand, and written informed consent was obtained. Participants included patients of all ages and both genders with clinical features suggestive of bone tumors or tumor-like lesions, recruited from outpatient and inpatient services of orthopedics, surgery, oncology, pediatrics, and medicine. Clinically and radiologically suspected bone lesion patients who underwent biopsy or surgical intervention with tissue submitted for histopathological examination were included. In contrast, non-pathological fractures, inoperable lesions, inadequate or poorly preserved specimens, non-compliance with radiological evaluation, prior chemo-radiotherapy, and hematological malignancies involving bone marrow were excluded.

The minimum sample size of 102 was determined using the formula $\, n = \frac{Z^2 p q}{d^2} \,$, where n represents the required sample size, Z is the standard normal deviate for a 95% confidence interval, p denotes the estimated prevalence (6%), q = 1 − p, and d represents the allowable margin of error (10%), considering the rare nature of bone tumors and limited case availability at the study center. A 10% non-response rate was applied. The calculation was based on departmental records indicating approximately 60 bone tumor biopsies annually. Consecutive sampling was used, and all eligible patients presented during the 18-month study period were included, resulting in a final sample size of 125. An estimated prevalence of 6% was derived from epidemiological studies [6]. All radiological evaluations and histopathological examinations were carried out as part of routine clinical management, and patients who met the eligibility criteria were subsequently recruited into the study and assigned unique identification numbers to ensure confidentiality. Clinical assessment was conducted using a pre-developed case record form that documented demographic information, presenting complaints, symptom duration, and relevant history. Radiological studies, including plain radiographs, computed tomography, and magnetic resonance imaging, were evaluated. Lesions were assessed based on anatomical site, nature, cortical involvement, periosteal reaction, matrix mineralization, and soft tissue extension, and a provisional clinico-radiological diagnosis was documented prior to histopathological examination.

Specimens were processed, and gross examination assessed size, morphology, tumor extent, and margins. Tissues were fixed in 10% neutral-buffered formalin, decalcified where necessary, and processed routinely for paraffin embedding. Sections of 4-5 µm thickness were stained with hematoxylin and eosin. Microscopic analysis evaluated cellular morphology, matrix production, mitotic activity, necrosis, and tumor margins. The final diagnosis was made according to the World Health Organization classification [12], and two pathologists reviewed all cases. Any disagreement between the two pathologists was to be resolved by consensus. However, no major interobserver disagreement affecting the final diagnosis was noted during the study. Comparisons between clinico-radiological diagnoses and histopathological findings were made and classified as concordant or discordant. Data were analyzed using SPSS Statistics version 27 (IBM Corp. Released 2020. IBM SPSS Statistics for Windows, Version 27.0. Armonk, NY: IBM Corp.). Cohen’s kappa statistic was used to assess agreement between radiological and histopathological diagnoses, and a p-value <0.05 was considered significant.

## Results

The mean age of the participants was 23.52 ± 8.10 years. Most patients were below 30 years of age, accounting for 98 (78%) cases, while 27 (22%) patients were aged 30 years or above. Among the 125 participants, 72 (58%) were male, and 53 (42%) were female, with a male-to-female ratio of 1.4:1. Rural and urban distributions were nearly comparable, accounting for 65 (52%) and 60 (48%) cases, respectively. Educational status was distributed across the categories: graduate (55, 44%), secondary education (40, 32%), and primary education (30, 24%).

**Figure 1:**
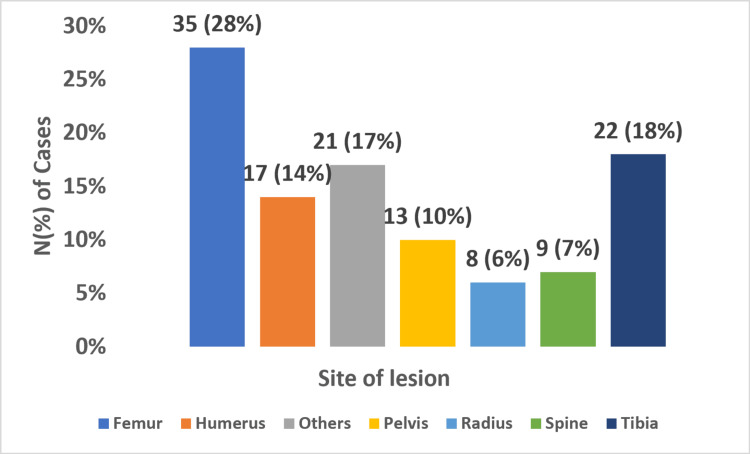
Distribution of lesions based on the site of occurrence (n = 125)

Lower socioeconomic status (modified BG Prasad classification) accounted for 58 (46%) cases, followed by middle socioeconomic status (48, 38%) and upper socioeconomic status (20, 16%). Family history was present in eight (6%) participants. Swelling with pain (48, 38%) was the most common presenting complaint, followed by pain alone (35, 28%) and swelling associated with pain and pathological fracture (21, 17%). The mean duration of symptoms was 5.57 ± 3.28 months. The distribution of bones showing pathological fracture is given in Figure 1.

Radiological assessment showed a heterogeneous variety of lesions, with the most common suspected diagnoses including chondroblastoma (18, 14%), enchondroma (16, 13%), and osteosarcoma (15, 12%). In general, malignant lesions were the largest radiological category, accounting for 52 (42%) cases, followed by benign lesions with 45 (36%), tumor-like lesions with 20 (16%), and metastatic lesions with 8 (6%) (Table 1).

**Table 1:** Categorization based on radiological diagnosis

Radiological diagnosis	N	%
Benign	45	36%
Malignant	52	42%
Metastasis	8	6%
Tumor-like lesion	20	16%
Total	125	100%

On histopathological examination, osteosarcoma was the most common malignant tumor, and osteochondroma, enchondroma, giant cell tumor, and chondroblastoma were the most common benign lesions. Malignant lesions represented 47 (38%) cases, while benign lesions accounted for 58 (46%) cases (Table 2).

**Table 2:** Categorization based on histopathological diagnosis

Histopathological diagnosis	N	%
Benign	58	46%
Malignant	47	38%
Metastasis	12	10%
Tumor-like lesion	8	6%
Total	125	100%

Overall concordance between radiological and histopathological diagnoses was observed in 106 (85%) cases, while 19 (15%) cases showed discordance, indicating moderate agreement between the two diagnostic modalities (Table 3).

**Table 3:** Concordance between radiological and histopathological findings

Concordance	N	%
No	19	15%
Yes	106	85%
Total	125	100%

Cross-tabulation analysis demonstrated concordant diagnoses in most benign and malignant lesions. However, a few cases of malignant and tumor-like lesions showed overlap between radiological and histopathological interpretations (Table 4).

**Table 4:** Cross-tabulation of radiology and histopathology

Cross-tabulation		Histopathological diagnosis											Total
		Aneurysmal bone cyst	Chondroblastoma	Chondrosarcoma	Enchondroma	Ewing sarcoma	Fibrous dysplasia	Giant cell tumor	Metastasis	Osteochondroma	Osteomyelitis	Osteosarcoma	
Radiological diagnosis	Aneurysmal bone cyst	4	0	0	0	0	0	1	0	0	0	0	5
	Bone infarct	0	0	0	1	0	0	0	0	0	0	0	1
	Brown tumor (hyperparathyroidism)	0	0	0	0	0	0	1	0	0	0	0	1
	Chondroblastoma	0	18	0	0	0	0	0	0	0	0	0	18
	Chondrosarcoma	0	0	8	0	0	0	0	0	0	0	0	8
	Enchondroma	0	0	1	15	0	0	0	0	0	0	0	16
	Ewing sarcoma	0	0	0	0	6	0	0	0	0	0	1	7
	Fibrous dysplasia	0	0	0	0	0	7	0	0	0	0	0	7
	Giant cell tumor	0	0	0	0	0	0	10	0	0	0	0	10
	Giant cell reparative granuloma	0	0	0	0	0	0	3	0	0	0	0	3
	Intraosseous ganglion cyst	0	0	0	0	0	0	1	0	0	0	0	1
	Low-grade chondrosarcoma	0	0	0	1	0	0	0	0	0	0	0	1
	Lymphoma	0	0	0	0	1	0	0	2	0	0	0	3
	Metastasis	0	0	0	0	0	0	0	9	0	0	0	9
	Multiple myeloma	0	0	0	0	0	0	0	1	0	0	0	1
	Myositis ossificans	0	0	0	0	0	0	0	0	1	0	0	1
	Osteochondroma	0	0	0	0	0	0	0	0	13	0	0	13
	Osteomyelitis	0	0	0	0	1	0	0	0	0	1	0	2
	Osteosarcoma	0	0	0	0	0	0	0	0	0	0	15	15
	Paraosteal osteoma	0	0	0	0	0	0	0	0	1	0	0	1
	Subungual exostosis	0	0	0	0	0	0	0	0	1	0	0	1
	Telangiectatic osteosarcoma	1	0	0	0	0	0	0	0	0	0	0	1
Total		5	18	9	17	8	7	16	12	16	1	16	125

The kappa value was 0.636 (p = 0.001), indicating statistically significant moderate agreement between radiological and histopathological diagnoses. Among the 19 discordant cases, the most frequent mismatches involved giant cell tumor (six cases), which had been radiologically interpreted as an aneurysmal bone cyst (one case), a brown tumor (one case), a giant cell reparative granuloma (three cases), and an intraosseous ganglion cyst (one case). Additional discordant diagnoses included enchondroma misclassified as a bone infarct and a low-grade chondrosarcoma; Ewing sarcoma misinterpreted as lymphoma and osteomyelitis; metastatic lesions radiologically diagnosed as lymphoma and multiple myeloma; and one case of telangiectatic osteosarcoma radiologically considered an aneurysmal bone cyst (see Appendices).

## Discussion

Bone tumors and tumor-like lesions represent a heterogeneous group of disorders with overlapping clinical and radiological characteristics, often posing diagnostic challenges in routine practice. In the present analysis, most patients were younger than 30 years, with a mean age of 23.52 ± 8.10 years. Mohapatro et al., Eriten et al., Manjani et al., and Bhanu et al. also reported peak incidence during the second and third decades of life [13-16]. This age predilection may be related to rapid skeletal growth, increased osteoblastic activity, and higher metaphyseal vascularity during adolescence and early adulthood, particularly in osteosarcoma and Ewing sarcoma [17,18]. The male predominance observed in our study is consistent with epidemiological trends reported in previous Indian and international studies. Mohapatro et al. and Eriten et al. similarly reported male predominance [13,14]. Greater physical activity and hormonal influences may contribute to the higher frequency among males [17-19]. Swelling associated with pain was the most common presenting symptom in our patients, followed by isolated pain. Similar observations were reported by Mohapatro et al. and Eriten et al. in their studies of bone tumors [13,14]. Pathological fractures in a minority of patients indicated aggressive tumor behavior or cortical involvement. The femur was the most commonly involved bone, followed by the tibia and humerus. Manjani et al. and Bhanu et al. also observed predominant involvement of long bones [15,16]. Previous studies have shown a characteristic metaphyseal predilection of primary bone tumors around the knee joint, where rapid skeletal growth and increased vascularity may promote tumor development, leading to predominant involvement of the femur and tibia [20].

Forty-two percent of lesions were classified as malignant on radiological evaluation, compared with 38% on histopathology, indicating a tendency toward radiological overestimation of aggressive lesions. Eriten et al. and Shojaie et al. reported similar findings in their analyses of bone tumors. Imaging features such as cortical destruction, periosteal reaction, and soft tissue extension may also be observed in aggressive benign or inflammatory lesions, thereby limiting radiological specificity [14,21]. This highlights the need for histopathological confirmation prior to definitive treatment. Histopathological examination revealed benign lesions in 46% of cases and malignant lesions in 38% of cases. Osteosarcoma was the most common malignant tumor, whereas osteochondroma, enchondroma, giant cell tumor, and chondroblastoma represented the predominant benign lesions. Similar findings were reported by Mohapatro et al. and Bhanu et al., who also identified osteosarcoma as the most common malignant bone tumor [13,16]. Histopathology remains the diagnostic gold standard because it enables detailed evaluation of tissue architecture, cellular atypia, mitotic activity, and matrix production [10,11].

Our study showed an overall concordance of 85% between radiological and histopathological diagnoses, with a kappa value of 0.636 (95% CI: 0.553-0.745), indicating statistically significant agreement (p < 0.001). Eriten et al. reported a concordance of approximately 84%, while Bhanu et al. observed a concordance approaching 87% [14,16]. High radiology-histopathology concordance supports the utility of imaging as an effective preliminary diagnostic modality for surgical planning and lesion stratification, particularly in resource-constrained tertiary centers. Despite substantial agreement, discordance was observed in 15% of cases, primarily due to overlapping imaging appearances and limited radiological specificity [20,21]. Benign aggressive lesions such as giant cell tumors and aneurysmal bone cysts may mimic sarcoma radiologically, whereas low-grade malignancies may occasionally demonstrate deceptively benign imaging features.

In resource-limited settings such as eastern India, delayed presentation, limited access to advanced imaging modalities, and socioeconomic constraints may further complicate timely diagnosis and management of bone lesions. Under such circumstances, integration of clinical evaluation, radiological assessment, and histopathological examination becomes particularly important to minimize diagnostic delay and avoid inappropriate treatment. The study was limited by its single-center design, relatively small sample size, and lack of advanced molecular or immunohistochemical correlation in selected lesions. Additionally, our study did not stratify discordance by radiological modality, such as X-ray, CT, or MRI.

## Conclusions

Bone tumors and tumor-like lesions mostly involved relatively younger individuals and commonly affected long bones, particularly the femur and tibia. Radiological evaluation showed high concordance with the histopathological diagnosis, although malignancy was overestimated in selected cases due to overlapping imaging features. Among malignant tumors, osteosarcoma was the most common, whereas osteochondroma, enchondroma, giant cell tumor, and chondroblastoma were the most common among benign lesions. Radiological evaluation demonstrated moderate agreement with histopathological diagnosis and served as an effective preliminary diagnostic modality in bone tumors and tumor-like lesions. These findings further emphasize the importance of imaging as a low-cost and reliable first-line diagnostic tool. However, histopathological examination remains necessary for confirmation and proper management.

## Appendices

**Table 5:** Radiological diagnosis as per individual lesion (n = 125)

Radiological diagnosis	N	%
Bone infarct	1	1%
Brown tumor (hyperparathyroidism)	1	1%
Intraosseous ganglion cyst	1	1%
Low-grade chondrosarcoma	1	1%
Multiple myeloma	1	1%
Myositis ossificans	1	1%
Osteomyelitis	2	2%
Giant cell reparative granuloma	3	2%
Lymphoma	3	2%
Aneurysmal bone cyst	5	4%
Ewing sarcoma	7	6%
Fibrous dysplasia	7	6%
Chondrosarcoma	8	6%
Metastasis	9	7%
Giant cell tumor	10	8%
Osteochondroma	13	10%
Enchondroma	16	13%
Chondroblastoma	18	14%
Osteosarcoma	15	12%
Paraosteal osteoma	1	1%
Subungual exostosis	1	1%
Telangiectatic osteosarcoma	1	1%
Total	125	100%

**Table 6:** Histopathological diagnosis as per individual lesion (n = 125)

Histopathological diagnosis	N	%
Aneurysmal bone cyst	5	4%
Chondroblastoma	18	14%
Chondrosarcoma	9	7%
Enchondroma	17	14%
Ewing sarcoma	8	6%
Fibrous dysplasia	7	6%
Giant cell tumor	16	13%
Metastasis	12	10%
Osteochondroma	16	13%
Osteomyelitis	1	1%
Osteosarcoma	16	13%
Total	125	100%

**Table 7:** Cross-tabulation of radiology and histopathological diagnosis

Cross tabulation		Histopathological diagnosis											Total
		Aneurysmal bone cyst	Chondroblastoma	Chondrosarcoma	Enchondroma	Ewing sarcoma	Fibrous dysplasia	Giant cell tumor	Metastasis	Osteochondroma	Osteomyelitis	Osteosarcoma	
Radiological Diagnosis	Aneurysmal bone cyst	4	0	0	0	0	0	1	0	0	0	0	5
	Bone infarct	0	0	0	1	0	0	0	0	0	0	0	1
	Brown tumor (hyperparathyroidism)	0	0	0	0	0	0	1	0	0	0	0	1
	Chondroblastoma	0	18	0	0	0	0	0	0	0	0	0	18
	Chondrosarcoma	0	0	8	0	0	0	0	0	0	0	0	8
	Enchondroma	0	0	1	15	0	0	0	0	0	0	0	16
	Ewing sarcoma	0	0	0	0	6	0	0	0	0	0	1	7
	Fibrous dysplasia	0	0	0	0	0	7	0	0	0	0	0	7
	Giant cell tumor	0	0	0	0	0	0	10	0	0	0	0	10
	Giant cell reparative granuloma	0	0	0	0	0	0	3	0	0	0	0	3
	Intraosseous ganglion cyst	0	0	0	0	0	0	1	0	0	0	0	1
	Low-grade chondrosarcoma	0	0	0	1	0	0	0	0	0	0	0	1
	Lymphoma	0	0	0	0	1	0	0	2	0	0	0	3
	Metastasis	0	0	0	0	0	0	0	9	0	0	0	9
	Multiple myeloma	0	0	0	0	0	0	0	1	0	0	0	1
	Myositis ossificans	0	0	0	0	0	0	0	0	1	0	0	1
	Osteochondroma	0	0	0	0	0	0	0	0	13	0	0	13
	Osteomyelitis	0	0	0	0	1	0	0	0	0	1	0	2
	Osteosarcoma	0	0	0	0	0	0	0	0	0	0	15	15
	Paraosteal osteoma	0	0	0	0	0	0	0	0	1	0	0	1
	Subungual exostosis	0	0	0	0	0	0	0	0	1	0	0	1
	Telangiectatic osteosarcoma	1	0	0	0	0	0	0	0	0	0	0	1
Total		5	18	9	17	8	7	16	12	16	1	16	125
